# The Book World of Medicine and Science

**Published:** 1893-12-09

**Authors:** 


### The Book World of Medicine and Science.

### BOOK REVIEWS.

A Manual of Medical Treatment or Clinical Therapeutics. By I. Burney Yeo, M.D. (Cassell and Co., London. 1893. Two vols., 21s.)

We do not know of any book like the one at present before us, for it is really a treatise on medicine in which the greater part of the description of each disease is occupied with an account of the treatment of it.

The title, we must confess, appears to us to be rather strange, for we fail to see how any branch of therapeutics can exist which is not clinical, and if this is so, the word clinical might be omitted. Also, the title conveys the impression that surgical treatment is not clinical therapeutics.

There are clearly two parts to be reviewed; one the therapeutical, and the other such of the account of each disease as does not deal with the treatment of it.

The therapeutic part is, on the whole, admirable, and the author is judicial in his praise of any particular mode of treatment, but much that the reader would like to know is left out. For example, in a book of this size, we should expect to find the composition of the commoner mineral waters, and fuller reference to many dietetic points that are only touched upon. Much space might have been saved by omitting all description of surgical operations, then we might have had fuller information about what is commonly called "general therapeutics," and instead of numerous references to other works by Dr. Burney Yeo, we might have found valuable extracts from them.

The first part deals with the treatment of diseases of the organs of digestion, and the directions given are eminently reasonable. We agree with most of Dr. Yeo's recommendations; nevertheless, we doubt very much his statement made on page 41 that the rectum can only absorb predigested substances, for we have seen patients kept alive for weeks on nutrient enemata which were not predigested. Also we feel sure he is wrong in stating that nutrient suppositories are not absorbed. We hardly think it necessary to submit patients to the disagreeable process of washing out the stomach if they are only suffering from a mild gastric catarrh. Among his prescriptions for gastritis we do not like that on page 57, which contains subnitrate of bismuth and bicarbonate of sodium, for the nitric acid in the bismuth compound often liberates carbonic acid gas from the bicarbonate, with the result that the cork is blown out of the bottle.

Part II. contains a description of the treatment of diseases of the heart, blood-vessels, blood, and ductless glands. It struck us that here the author laid too much stress on tobacco as a cause of cardiac dilatation, and that in giving an ounce of infusion of digitalis every six hours he was advising rather too large a dose. Among the many drugs recommended for the sleeplessness so often associated with heart disease, we think chloramide should have been mentioned, for it is a most valuable drug. The author's account of the treatment of fatty heart is good, and his remarks on the "Terrain Kur" are particularly just and sensible.

Turning now to such of the first volume as does not deal with treatment, we think that it is far inferior; in fact, in many cases the teaching is in our opinion unsupported by facts, and throughout one cannot help feeling that perhaps the writer has not a wide acquaintance with appearances

### The Hospital Advertiser.

THE BOOK WORLD OF MEDICINE AND SCIENCE—*continued.*

seen in the dead-house, and after all, without the knowledge that can only be gained there, medicine cannot be properly understood. As instances of what we mean, we may mention the statement that gastric ulcer is commoner in women than in men. This is by no means certain, for in the dead-house it is more frequently found in men than in women, a fact that makes it possible that clinically our diagnosis is often wrong. Then the statement (p. 169) that "a morbid state of the sympathetic nerve trunks" appears to be the cause of enteralgia is not medicine, but vague speculation without, as far as we know, any trustworthy histological evidence. On page 226 the author distinctly rejects the evidence afforded by post-mortem examinations, that nearly all cases of perityphlitis are due to disease of the appendix, preferring to trust to clinical evidence, although it is notorious that post-mortem examinations are, in abdominal complaints, constantly proving the diagnosis to be wrong. Again, we hardly think the author is wise in stating that rheumatic peritonitis does occur, without giving anatomical evidence, considering how frequently causes for peritonitis unsuspected during life are found post-mortem. Also we should like some proof from the author's own experience of the statement that cancer, gout, and diabetes can produce simple endocarditis. In describing pernicious anæmia, he does not mention the increase of free iron in the liver, but does state that the urine contains pathological urobilin, although all the more recent authorities deny the existence of this body. It appears to us that the descriptions of colitis, cæcitis, and appendicitis are unsupported by post-mortem evidence. Ulcerative colitis, a distinct disease, the existence of which would never have been known but for post-mortem examinations, is left out altogether.

We have read the second volume carefully, but there is nothing in it that calls for comment after what we have said of the first.

We have found very few misprints. The book can hardly be said to be interesting, but that is the fault of the subject, for it would be very difficult for anyone to make a book containing so many prescriptions anything but dull.

### DECEMBER REVIEWS.

Mrs. Percy Frankland has another of her instructive little papers in Longman's Magazine on water bacteriology, pointing out the overwhelming importance of sand filtration, illustrated by the cholera epidemic in Hamburg and Altona. Both of these cities are dependent on the Elbe for their water supply. Hamburg receives the water in a comparatively pure state from an intake above the city and made no provision for filtration. Altona gets the water from below Hamburgh after it has received the sewage of nearly 800,000 persons and purifies it by means of filter beds, the result being that in one street cholera was rampant in those houses supplied with Hamburg water, while in those on the Altona side of the way, supplied with Altona water, not one case occurred. The importance of keeping the sand filters under continual inspection is shown by the fact that a sudden recrudescence of cholera in the city of Altona was traced not without some difficulty to the freezing of one of the filters which was thus rendered ineffectual for retaining the bacteria.

### The Hospital Advertiser.

### THE BOOK WORLD OF MEDICINE AND SCIENCE—(*continued*).

MR. HUGH PERCY DUNN pursues an interesting inquiry in the NINETEENTH CENTURY into "What London People Die Of?" No one will be surprised to learn that taking the mortality rates in order of precedence, diseases of the respiratory organs come first on the list. Chief among the victims to bronchitis and pneumonia are the little children who perish in thousands from these causes within the first five years of life. In diseases of the nervous system, which hold the second place, it is curious to see that London compares very favourably indeed with the rest of England, and the facts produced serve to demonstrate that the constant stimulus of the City life is less wearing in its effects than has generally been supposed. In diseases of the circulatory system and of the digestion Londoners are able also to show an excellent record compared with dwellers in other places; both their hearts and stomachs show a more than average condition of well-being. It is their lungs which are principally in danger. Making due allowance for those persons who come up from the country to be treated for phthisis "it is impossible to come to any other conclusion than that in London the surroundings are pre-eminently favourable to the dissemination of tuberculous affections." When the disease is once fairly recognized as contagious, and not till then, can a definite improvement be expected.

IN the CONTEMPORARY REVIEW the Spencer-cum-Weissmann controversy continues, with the very instructive result of showing "what entirely opposite conclusions men may draw from the same evidence." Labour questions absorb a large part of the number this month, which is full of thoughtful and suggestive reading.

IT would be difficult to lay one's hand on a more naive collection of truisms than those Dr. Robson Roose has put together for the readers of the FORTNIGHTLY REVIEW in his article on Clothing as a Protection against Cold. "Garments made of pure silk are exceedingly comfortable but very expensive. Furs and leather are serviceable against great cold. Waterproof clothing should be reserved for very wet weather. The source of heat is within the body itself and not in the clothes." These are some of the writer's striking discoveries. The unedited letters of Keats which Mr. Forbes Sieveking has unearthed are strangely unworthy of their late resurrection, containing every fault of his careless correspondence prose, with scarcely a glimpse of self-revelation.

THE NEW REVIEW has an appreciative article on M. Charcot by Mdlle. Blaze de Bury, and a protest by Mr. Frederick Boyle against the Decay of Beauty in the present age, which he attributes partly to the fact that the moderns wear too much clothing, and partly to the fact that they spend too little time in their baths.

